# A postmortem case control study of asbestos burden in lungs of malignant mesothelioma cases

**DOI:** 10.1186/s12967-023-04761-9

**Published:** 2023-12-02

**Authors:** S. D. Visonà, B. Bertoglio, C. Favaron, S. Capella, E. Belluso, C. Colosio, S. Villani, T. Ivic-Pavlicic, E. Taioli

**Affiliations:** 1 Department of Public Health, Experimental and Forensic Medicine, Unit of Legal Medicine and Forensic Sciences, University of Pavia, Pavia, Italy; 2 Department of Biology and Biotechnology “L. Spallanzani”, University of Pavia, Pavia, Italy; 3 Department of Earth Sciences, University of Torino, Turin, Italy; 4 Interdepartmental Center for Studies on Asbestos and other Toxic Particulates “G. Scansetti”, University of Torino, Turin, Italy; 5 Department of Health Sciences, University of Milan, Milan, Italy; 6 Occupational Health Unit, Santi Paolo e Carlo Hospital, Milan, Italy; 7 Department of Public Health, Experimental and Forensic Medicine, Unit of Biostatistics and Clinical Epidemiology, Pavia University, Pavia, Italy; 8 Institute for Translational Epidemiology and Tisch Cancer Institute, Icahn School of Medicine at Mount Sinai, New York, NY USA

**Keywords:** Malignant mesothelioma, Asbestos, SEM–EDS, Autopsy, Chrysotile, Amphiboles, Epidemiology

## Abstract

**Background:**

Asbestos lung content is regarded as the most reliable tool for causal attribution of malignant mesothelioma (MM) to previous asbestos exposures. However, there is a lack of studies on asbestos burden in lungs of MM patients in comparison with healthy individuals. This study aims to provide such a comparison, investigating, as well, differences in asbestos lung burden with sex and time trends.

**Methods:**

Asbestos lung content has been assessed on formalin-fixed lung fragments using scanning electron microscopy coupled with energy dispersion spectroscopy (SEM–EDS) on individuals deceased from MM (cases) and healthy subjects without any lung disease who died from violent causes (controls) between 2005 and 2023.

**Results:**

Asbestos and asbestos bodies (ABs) were found, respectively, in 73.7% and 43.2% of cases and in 28 and 22% of controls; in MM cases the most represented asbestos types were crocidolite and amosite, whereas in controls it was tremolite-actinolite asbestos. The concentration of both asbestos fibers and ABs was statistically significantly higher in MM cases compared to controls. The mean asbestos fibers width was also significantly higher in cases than controls. Males and females with MM showed similar asbestos and ABs concentrations, but females had higher concentrations of chrysotile, and significantly lower fibers width compared to males. Time trends show that MM lung asbestos concentrations decreased starting in 2011.

**Discussion:**

The results suggest a correlation between asbestos burden in lungs and MM risk. The different concentration of chrysotile, as well as the different width of asbestos fibers in MM males and females might reflect a sex difference in response of the lung microenvironment to inhaled asbestos. Finally, this study provides the first pathological evidence of the effect of the ban of asbestos use, demonstrating a significant decrease of asbestos lung content after 2011.

**Supplementary Information:**

The online version contains supplementary material available at 10.1186/s12967-023-04761-9.

## Background

Asbestos is a term comprising six naturally occurring fibrous minerals: chrysotile (the only one belonging to serpentines), the commercial amphiboles crocidolite, amosite and anthophyllite asbestos, as well as the non-commercial tremolite and actinolite asbestos [1]. All types of asbestos are well known to cause diseases in humans and animals, both benign (pleural plaques and lung fibrosis) and malignant, such as malignant mesothelioma (MM) and lung cancer [2]. Asbestos-related diseases, especially MM, still represent a major public health concern. In fact, despite the total ban of asbestos implemented in the 90 s in most European countries and the strict regulation introduced in US and Canada, this mineral is still present in our environment, as shown by studies conducted on air and, more recently, on lungs obtained from otherwise healthy deceased subjects from the general population [3–5]. Moreover, as MM is characterized by a long latency (30–40 years), its onset can be traced back to exposures that occurred and often ceased decades ago [1]. For this reason, in Italy as well as in other countries, the peak of MM incidence has not been reached yet, and it is expected to occur in the next few years [6].

Despite the numerous studies about asbestos lung content [7–9], the effect of asbestos concentration and type on MM development is still not fully understood, as there are few studies that compare asbestos lung content in MM patients and controls [10–12]. The few existing studies have been carried out several years ago, when asbestos was certainly more widely diffused than today, and do not reflect the current situation in Europe. Moreover, in Howel and al. [11] the cause of death of controls was attributed to various diseases, and this can introduce a bias. The present study, instead, allows to measure the lung content in healthy deceased subjects without any history of asbestos exposure or respiratory diseases, and to compare it to MM cases, in order to quantify the amount of “background exposure”.

Asbestos lung content is considered the most reliable way in order to establish a causal association with MM, especially in a legal context [13]. Therefore, it is of paramount importance to investigate the asbestos lung burden in healthy subjects from the general population, in order to quantify a threshold value that could differentiate between “background exposure” and exposure causally associated with MM. Since the Selikoff pioneer study that concluded that the amount of asbestos necessary to cause MM may be extremely low[14], there is no agreement in literature about the dose of inhaled asbestos that is necessary in order to cause MM; some studies found a correlation between asbestos lung burden and MM, whereas others did not [15]. In addition, little is known on whether lung content varies between males and females, given the known differences in type and length of exposure, and the differences in MM clinical and pathological characteristics between the two sexes [16, 17].

Building on these premises, the present work has four objectives: 1) to assess if the lung asbestos content (in terms of concentration of asbestos fibers) differs in subjects deceased from MM versus healthy controls without any known history of exposure to asbestos, and deceased from violent causes 2) to evaluate differences in ABs, dimensions, characteristics and concentrations of the individual asbestos types between cases and controls;to verify if sex and type of exposure can influence asbestos content in lungs; 3) to investigate the time trend of asbestos concentration in lungs.

## Materials and methods

### Study design, setting and participants

A case–control study was applied. The study will be carried out on autopsy material (epidemiological unit) from the archive of the Section of Forensic Medicine and Forensic Science (Department of Public Health, Forensic Experimental Medicine) of Pavia University.

Cases were selected among the autopsy material regarding subjects deceased for MM (inclusion criteria) and for which a forensic autopsy was performed at the Unit of Legal Medicine and Forensic Science of Pavia University between 2005 and 2018. The autopsy was followed by histopathological exams, including immunohistochemistry according to the guidelines in effect at the time [18–21].

In the vast majority, cases lived in Broni or in the hinterland of this small town in the Pavia Province (northern Italy), where the Fibronit factory was located. This factory was active between 1932 and 1993, and produced asbestos-cement artifacts using a mixture of chrysotile and amphiboles (mainly crocidolite, with small amounts of amosite [22, 23].

Controls were selected among otherwise healthy individuals deceased between 2001 and 2023 from traumatic causes and subjected to a forensic autopsy, followed by a complete histopathological exam, according to the following criteria:

- age above 40 years
- no medical history of neoplastic or respiratory disease
- no neoplastic or respiratory disease at postmortem examination
- living outside the area of Broni and surrounding municipalities and outside other areas known for significant sources of asbestos exposure
- negative known history for occupational, household or anthropogenic environmental asbestos exposure.

For each case, both lungs were formalin-fixed and stored, while for controls several formalin-fixed lung fragments were available from the archive.

### Variables, data sources and measurement

For each subject, a sample of 0.25 g of wet lung, taken from the inferior right lung lobe, was investigated with a scanning electron microscope equipped with energy dispersive spectroscopy (SEM-EDS) to assess the following endpoints:

- concentration of asbestos fibers, expressed as number of fibers per gram of dry weight (ff/gdw)
- mean length and width of detected asbestos fibers (expressed in μm)
- the concentration of each asbestos (ff/gdw), classified as chrysotile/asbestiform antigorite, crocidolite, amosite, tremolite/actinolite asbestos, and anthophyllite asbestos.
- concentration of asbestos bodies (ABs), expressed as ABs/gdw

In the present work only regulated asbestos fibers (length > 5 µm, width < 3 µm, aspect ratio greater than or equal to 3:1) [24] were counted, measured and classified according to the EDS spectrum.

Moreover, demographic characteristics (sex, age), history of exposure (occupational, household, environmental), duration of exposure in years, survival time in months, latency in years, were also evaluated.

#### SEM samples preparation

Formalin-fixed lung samples were washed in distilled water and then chemically digested using 13% sodium hypochlorite, then the suspension was filtered through a cellulose-ester membrane (Millipore, Darmstadt, Germany) with a diameter of 25 mm and a pore size of 0.45 µm as already described elsewhere [25, 26].

The membrane was then prepared for SEM–EDS examination and an area of 2 mm^2^ was observed at a magnification of 4000 × using backscattered electrons.

The fiber chemical composition was analyzed using an EDS, Oxford Inca Energy 200, equipped with an INCA X-act SDD detector (Oxford Instruments NanoAnalysis, Bucks, UK).

As indicated by international guidelines [28, 29], the amount of asbestos fibers and ABs observed in an area of 2 mm^2^ was normalized to 1 g of dry tissue, reporting concentration in terms of asbestos fibers and ABs per gram of dry weight of lung tissue: ff/gdw.

To identify the different types of inorganic fibers, we compared the EDS spectra with a reference database available in the laboratory that performed the tests.

SEM–EDS is not the appropriate approach to distinguish unequivocally chrysotile from asbestiform antigorite and tremolite from actinolite asbestos, since they have similar chemical composition and analogous morphology, therefore we used, respectively, the term chrysotile/asbestiform antigorite and tremolite/actinolite asbestos for these minerals.

While the preparation of all samples has been carried out in the same laboratory, the SEM EDS investigation was carried out in two laboratories, and the samples were divided equally between the two labs. In order to avoid the variability deriving from different instruments and microscopists, we defined a detailed, standardized protocol for data collection. A periodic inter-laboratory control was conducted by comparing the images and spectra obtained by each laboratory. In addition, five samples were analyzed in both laboratories, and the inter-laboratory variability was tested with ANOVA for repeated measurements [Additional file 1: Table S1].

### Statistical analysis

Quantitative variables were summarized by mean and standard deviation (SD) and qualitative with frequencies and percentage. Unpaired t-tests was applied to evaluate differences in asbestos fiber concentrations between MM cases and controls, or the analogous non parametric test if the assumptions were not verified. Secondary analyses comparing asbestos concentration in male with respect to female MM cases was conducted by means of the same test. The χ^2^ tests or Fisher’s Exact Test when > 25% of the expected cell counts were less than 5 was utilized for comparing categorical variables between cases and controls as well as males and females.

The adjusted effect of asbestos concentration for age and sex on MM were obtained using logistic regression. In the secondary analyses the effect of sex on asbestos concentration adjusted for age and type of exposure was obtained using multiple linear regression. Statistical significance was defined as* P* < 0.05. Statistical analyses were performed using SAS, version 9.4.

## Results

The study included 95 MM cases and 50 controls. The mean age at death was 69.57 years (SD 11.37 years) in MM cases and 72.32 years (11.25) in the controls (p = 0.17); 51.6% of MM cases were males, versus 60% among controls (p = 0.33).

MM patients reported occupational exposure in 38.9%, household in 17.9%, and anthropogenic environmental in 42.1% of cases; one case of MM had no history of exposure.

The median duration of exposure among cases was 23 years (IQR 14–31 years), the median survival time since the diagnosis was 13.5 months (IQR 9–20 years), and the median time elapsed between the end of exposure and death was 23 years (IQR 18–32 years).

Only two cases had peritoneal MM, while the other 93 (97.9%) had pleural MM. The MM histologic type was epithelioid in 73.7% of cases, sarcomatoid in 9.5%, biphasic in 15.8% and desmoplastic in 3.2%.

At SEM–EDS examination, asbestos fibers were identified in 73.7% of cases and in 28% of controls, whereas ABs in 43.2% and 22% respectively. In 15.8% of MM patients the asbestos concentration was between 1 and 9999 ff/gdw, in 43.2% between 10000 and 99999 ff/gdw, in 13.7% between 100000 and 999999 ff/gdw and in only 1 case (1%) asbestos concentration was above 1 million ff/gdw. Among controls, the corresponding figures were 18%, 10% and 0%.

Chrysotile was found in 6.3% of MM cases, crocidolite in 50.5%, amosite in 57.9%, anthophyllite asbestos in 4.2% and tremolite/actinolite asbestos in 53.7%. Among controls, chrysotile was observed in 6%, crocidolite in none, amosite and anthophyllite asbestos in only one case (2%) and tremolite/actinolite asbestos in 24%. The proportions of chrysotile and anthophyllite asbestos presence were not significantly different between cases and controls, (p > 0.90 and p = 0.6596, respectively). The proportions of crocidolite, amosite, and tremolite/actinolite were significantly different between cases and controls (p < 0.0001, p < 0.0001, and p = 0.0006, respectively).

When considering asbestos as a whole In MM patients 0.54% was represented by chrysotile, 40.58% by crocidolite, 48.33% by amosite, 0.85% by anthophyllite asbestos and 9.70% by tremolite/actinolite asbestos. In controls, 15.3% of asbestos was represented by chrysotile, 3.86% by amosite, 8.1% by anthophyllite asbestos and 72.7% by tremolite/actinolite asbestos. The distribution of the types of asbestos was significantly different between cases and controls (p < 0.0001).

The concentration of asbestos resulted significantly higher in cases with respect to controls (p = 0.0002), as well as the ABs concentration (p = 0.0137) (Table 1). The concentrations of amosite and tremolite/actinolite asbestos were significantly higher in MM patients compared to controls (p = 0.0051 and 0.0054 respectively) while the differences between the other types of asbestos did not reach statistical significance.

**Table 1:** Lung asbestos content in mesothelioma cases versus controls

	Mesothelioma n = 95 (65.52%)	Controls n = 50 (34.48%)	p-value	p-value ^
	Mean (standard deviation)			
Asbestos concentration (ff/gdw)	79705.59 (240333.83)	3187.74 (7582.45)	0.0025	0.0002
Concentration of asbestos bodies (ff/gdw)	74670.71 (333130.60)	2013.54 (5627.97)	0.0362	0.0137
Concentration of Chrysotile asbestiform antigorite (ff/gdw)	433.78 (1886.95)	488.85 (2131.73)	0.8734	0.8181
Concentration of crocidolite (ff/gdw)	32343.66 (102023.53)	0 (0)	0.0026	0.1557
Concentration of amosite (ff/gdw)	38522.37 (142833.63)	123.04 (870.05)	0.0102	0.0051
Concentration of anthophyllite asbestos (ff/gdw)	676.27 (3471.68)	257.27 (1819.19)	0.3419	0.3925
Concentration of tremolite actinolite asbestos (ff/gdw)	7729.52 (13009.02)	2318.58 (5979.66)	0.0008	0.0054
Mean length of asbestos fibers (μm)	23.87 (12.42)	19.87 (14.99)	0.2929	0.2749
Mean width of asbestos fibers (μm)	0.68 (0.28)	0.96 (0.38)	0.0027	0.0070
Survival (months)	17.40 (14.74)	–	–	–

^adjusted for age and sex with logistic regression

^#^fibers per gram of dry weight

Regarding asbestos dimensional characteristics, among cases the length ranged between 6 and 55 μm, with a mean of 23.87 μm, while the width ranged between 0.21 μm and 1.9 μm, with a mean of 0.68 μm. In controls the length of asbestos ranged between 5.4 μm and 52.4 μm, with a mean of 19.87 μm, while width ranged between 0.5 μm and 1.8 μm, with a mean 0.96 μm.

The length of asbestos did not show any statistically significant difference between cases and controls, while fibers were significantly wider in controls compared to cases (p = 0.0070) (Table 1).

### Characteristics of mesothelioma according to sex

Age at death, length of survival, latency and duration of exposure were not statistically different between sexes, while the type of exposure differed (Table 2). Namely, occupational exposure was more represented in males compared to females, whereas the opposite was observed for household and anthropogenic environmental exposure. The frequencies of each histologic type were similar between sexes.

**Table 2:** Characteristics of mesothelioma cases according to sex

	Male n = 49 (51.58%)	Female n = 46 (48.42%)	p-value
Age at death (years) Mean (SD)	70.65 (67.50)	68.41 (64.91)	0.3412
Latency (years) Mean (SD)	48.63 (9.84)	50.59 (15.67)	0.4817
Duration of exposure (years); Mean (SD)	22.15 (13.69)	27.32 (17.14)	0.1169
Survival (months) Mean (SD)	15.76 (11.79)	19.20 (17.37)	0.2682
History of exposure			
None	0 (0.00%)	1 (2.17%)	< 0.0001**
Occupational	33 (67.35%)	4 (8.70%)	
Household	4 (8.16%)	13 (28.26%)	
Anthropogenic/Environmental	12 (24.49%)	28 (60.87%)	
Histology			
Epithelial	37 (75.51%)	32 (69.57%)	
Sarcomatoid	3 (6.12%)	6 (13.04%)	0.5964**
Biphasic	8 (16.33%)	6 (13.04%)	
Desmoplastic	1 (2.04%)	2 (4.35%)	

^**^Fisher’s exact test

The concentration of asbestos and ABs were not significantly different between males and females when considering the p-value adjusted for age and type of exposure (Table 3). Among the asbestos types, only chrysotile was significantly more represented in females compared to males (p = 0.0187). Asbestos length was not significantly different between males and females, while width showed a tendency to be greater in males, without reaching statistical significance.

**Table 3:** Lung asbestos concentrations in mesothelioma cases according to sex

	Male n = 49 (51.58%)	Female n = 46 (48.42%)	p-value	p-value *
	Mean (standard deviation)			
Asbestos concentration (ff/gdw)	116718.43 (328061.05)	40278.87 (52680.63)	0.1138	0.7500
Concentration of asbestos bodies (ff/gdw)	138342.88 (456357.00)	6846.01 (23532.36)	0.0496	0.4412
Concentration of *Chrysotile asbestiform* antigorite (ff/gdw)	0 (0)	895.85 (2648.50)	0.0265	0.0187
Crocidolite Concentration (ff/gdw)	51355.28 (138066.69)	12092.15 (24429.16)	0.0557	0.5843
Amosite concentration (ff/gdw)	57712.94 (196478.55)	18080.24 (24713.63)	0.1677	0.8575
Anthophyllite asbestos concentration (ff/gdw)	1311.14 (4770.03)	0 (0)	0.0603	0.2537
Concentration of tremolite/ actinolite asbestos (ff/gdw)	6339.10 (11869.49)	9210.63 (14102.75)	0.2847	0.7146
Mean length of asbestos fibers (μm)	23.37 (13.97)	24.22 (11.33)	0.7808	0.4260
Mean width of asbestos fibers (μm)	0.80 (0.33)	0.60 (0.21)	0.0080	0.0763

^*^adjusted for age and type of exposure

^#^fibers per gram of dry weight

### Time trends

**Fig. 1:**
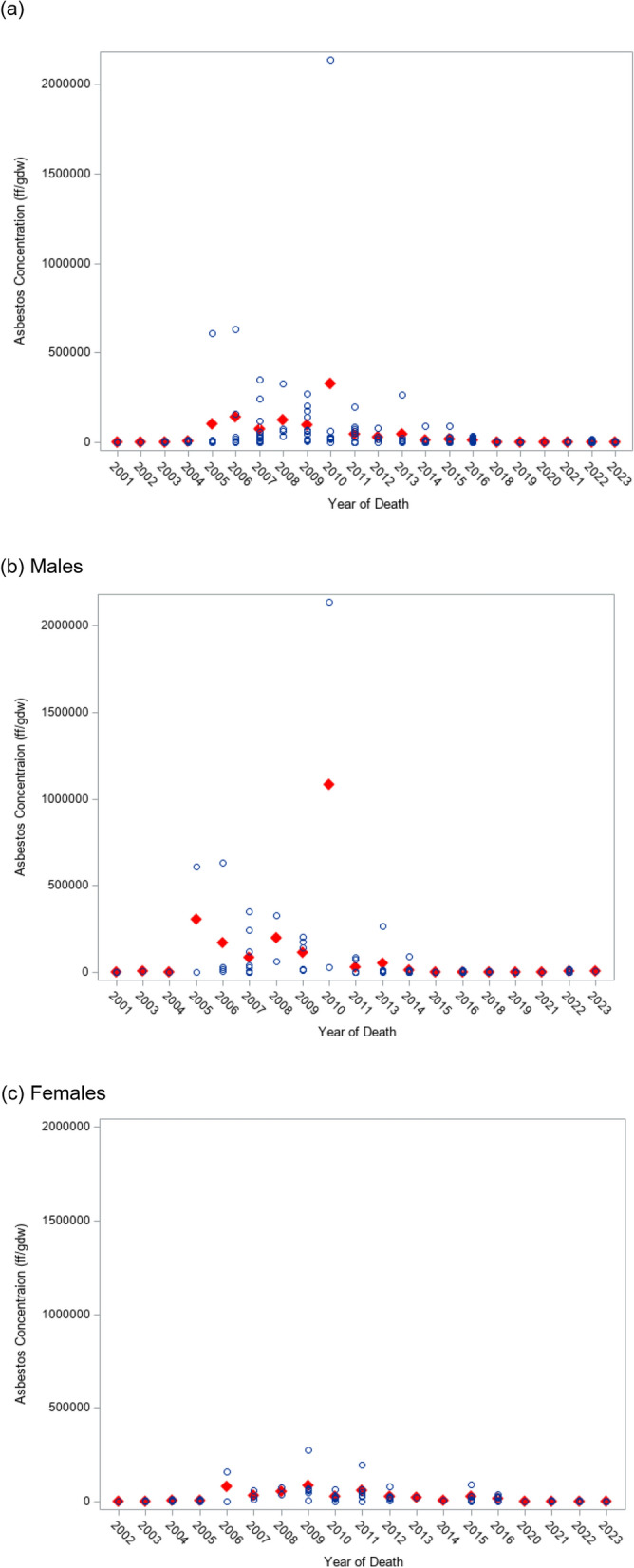
Average asbestos concentration (fibers per gram of dry weight), overall (**a**), and according to sex. **b** males; **c** females. Red diamonds indicate mean asbestos concentration (ff/gdw) per year

When lung asbestos concentrations were plotted against the year of death, we observed that asbestos lung concentrations decreased significantly after 2011 (Fig. 1), overall (**a**) and in males (**b**). Females (Fig. 1**c**) presented lower levels across all years, and showed a slight decrease in concentrations after 2012.

## Discussion

Overall, this study shows that asbestos is present in lungs of healthy individuals from the general population (controls), but in significantly lower concentrations compared to MM patients, confirming that asbestos concentration in lungs is positively related to MM occurrence.

We show here for the first time that sex does not seem to influence the total asbestos lung burden, even though females show a higher chrysotile concentration with respect to males. Moreover, survival since the diagnosis of MM was not significantly different between males and females in our sample. Finally, time trends demonstrated, overall and in males, a significant decrease of asbestos lung burden after 2011. In females the levels of asbestos were lower across the period of time considered and showed a slight decrease in concentrations after 2012.

One of the main limitations of this study is that only asbestos fibers longer than 5 µm, thinner than 3 µm, and with an aspect ratio greater than or equal to 3:1) [24] have been systematically counted and measured at SEM–EDS examination. However, some authors reported a relationship between fibers with a low aspect ratio and MM, and attributed this to the fact that their smaller size allows them to reach the pleural cavity more easily [7, 27]. However, the widely shared opinion is that fibers longer than 10 μm play the most important role in MM and lung cancer causation [1, 28, 29].

A second limitation relates to ABs. Here we analyzed them at SEM–EDS, in the same sample we used to assess uncovered asbestos fibers. That is not the most suitable technique for examining ABs, as usually they are quantified using a larger lung fragment and under light microscope (possibly in combination with SEM), in order to observe a larger area of the filter [30]. Therefore, the concentration of ABs in this study may be underestimated.

A third possible limitation is related to the single sampling site. The inferior lobe of the right lung, based on anatomic and physiological reasons, has long been known as the area where a larger number of particles accumulate [31]. For this reason, and also due to random factors, there is a remarkable variability between different lung areas [32, 33]. Therefore, for clinical and forensic purposes it would be better to analyze multiple samples. However, within the same study it is important to analyze lung parenchyma taken from the same area in order not to introduce a potential confounding factor.

Despite these limitations, we can report here that asbestos and ABs were identified in both cases and controls. Interestingly, in 26.3% of MM patients did not have asbestos in their lung samples. In addition, the majority of MM cases presented an asbestos lung content below 100000 ff/gdw, that is the threshold value (determined using SEM–EDS), for amphiboles, considered to be indicative of a significant past exposure to asbestos [20]. The finding of an asbestos concentration lower than expected in MM patients with documented history of occupational, household and/or anthropogenic environmental exposure is similar to what was found in previous studies by our group [26, 34]. This may be the result of asbestos clearance in the lungs, a known phenomenon mainly for chrysotile, due to its crystalline structure that, unlike amphiboles, can be fragmented in the lung microenvironment, phagocyted and eventually removed by macrophages through the lymphatic system [35–38]. Consequently, the finding of low concentrations of chrysotile and in some cases, the total absence of asbestos in MM patients is not surprising, especially considering the remarkable period of time elapsed between the cessation of exposure and death in the patients included in this study.

On the other hand, 28% of healthy subjects whose anamnesis was negative for any known asbestos exposure, were positive for asbestos at SEM–EDS examination, although at concentrations below the threshold of 100000 ff/gdw, confirming the validity of this cut-off value to identify past asbestos exposures. Thus, the present data suggest that asbestos concentration on lungs above this value are a strong sign of past occupational, household or anthropogenic environmental exposure to asbestos, but the opposite cannot be stated. The concentration of ABs, both in cases and controls, showed a large variability (from 0 to more than 7 million ABs in MM and from 0 to 30600 in controls). It is interesting to note that in a remarkable number of controls the concentration of ABs was well above the 1000 ABs/gdw suggested by Churg as a “break point” between the non-occupational and occupational levels of exposure [39], similarly with what was found by Case et al. in a series of forensic autopsies [40].

The concentration of both asbestos fibers and ABs was significantly higher in cases compared to controls, as expected. This finding is in contrast with some studies comparing lung content in MM patients and “controls” [41], while other authors found similar significant differences as we report here [10, 11]. One caveat is that most of the previous studies were conducted in the 70 s and 80 s, when asbestos-containing materials were extremely diffused and when previous asbestos exposures, even if unknown or forgotten, were very likely. In contrast, the present series of controls were selected among autopsies performed in recent years, well after the asbestos ban in Italy (which occurred in 1992).

Notwithstanding, the significant difference in asbestos concentration between MM and controls, confirms the well-established relationship between asbestos and MM, and supports the hypothesis that asbestos concentration can influence the risk of developing MM [42–44].

Even though the concentration of the individual asbestos types did not differ significantly between cases and controls, in MM the majority of asbestos was classified as crocidolite and amosite, while in controls asbestos was mostly represented by chrysotile and, especially, tremolite-actinolite asbestos. This is in line with what is known about past exposure in these cases and controls: cases were exposed mainly in relation to an asbestos cement plant, where large amounts of chrysotile, crocidolite and amosite were used in manufacturing asbestos-containing artifacts. Instead, asbestos found in lungs of healthy individuals is likely to derive from urban pollution [3, 4] or talc-containing products [45, 46].

When we compared males and females, the two sexes presented a different history of exposure. As previously known, males are generally exposed mainly occupationally, while females through a family member or environmentally [16, 47, 48]. Despite this different exposure setting, asbestos and ABs concentrations did not show any statistically significant difference between the two sexes. This underlines the relevance of non-occupational exposure, which can determine asbestos lung levels comparable to the occupational one, as already pointed by both epidemiological data [49, 50] and lung content analysis [47, 51].

In addition, the comparison between males and females pointed out that chrysotile is significantly more represented in females’ lungs. This is relevant, as the lower carcinogenic potential of chrysotile in humans is related to its rapid clearance compared to amphiboles [28, 35, 52]. This novel finding might indicate a different reaction to chrysotile in the lung microenvironment of males and females that can change the amount of asbestos reaching the pleural cavity and consequentially the carcinogenicity potential of chrysotile. The tendency of males to have wider fibers compared to females is in line with this hypothesis, as a higher concentration of chrysotile may be related to a more efficient longitudinal splitting of chrysotile bundles and therefore to thinner fibers [53].

Moreover, although females are known to have a better prognosis after MM diagnosis [17], in the present study the survival did not show any significant differences according to sex. This could be possibly related to the females’ heavy asbestos burden, similar to what observed in males in our study, whereas in other series lung burdens in females might have been lower, in relation to their more frequent non-occupational exposure. In fact, asbestos burden in lungs has previously been related to MM prognosis by some authors [54], even though the opposite has been found in another study [55]. Moreover, the better survival in females had been previously also related to the more frequent epithelioid histology in females [17], whereas in this study the frequency of each histologic type, did not show any clear tendency.

Finally, asbestos lung burden showed a decrease after 2011 (overall and in males) and after 2012 in females. This trend may reflect the effect of the restrictions and improvements in safety measures introduced in the 80 s and, more importantly, of the asbestos ban, introduced in Italy in 1992. This is the first pathological evidence of the effect of the ban of asbestos, in line with the decrease in MM incidence pointed out recently in most countries that introduced a ban of asbestos, as well as in Australia [56, 57]. The different trend in females seems to reflect their different type of exposure, mostly environmental, that, unlike occupational exposure, did not disappear completely after asbestos ban.

## Conclusions

In the present article, we investigated the relevance of asbestos lung burden in determining MM risk by comparing deceased MM patients to the general population. In addition, we assessed possible differences in asbestos lung content between males and females.

The results suggested that asbestos concentration is significantly lower in controls (and namely always below 100000 ff/gdw) compared to MM cases. Secondly, we found that females have a higher concentration of chrysotile and thinner fibers compared to males, and this might reflect a different response to chrysotile in the lung microenvironment.

This study adds meaningful and novel insights in the role of asbestos in MM, as it is one of the very few, in literature, that investigates lung content not only in cases of asbestos-related diseases, but also in a carefully selected series of appropriate controls. Moreover, the novel findings about possible differences in asbestos handling inside the lung microenvironment between males and females open new perspectives in the understanding of the carcinogenic potential of asbestos and calls for more research in this field. Finally, this study provides the first pathological evidence of the effect of the ban of asbestos twenty years after its implementation, demonstrating a significant decrease of asbestos lung content after 2011.

### Supplementary Information

**Additional file 1: Table S1.** Reliability of measurements performed in two different laboratories.

## Data Availability

The datasets used and/or analysed during the current study are available from the corresponding author on reasonable request.

## References

[1] Carbone M, Adusumilli PS, Alexander HR, Baas P, Bardelli F, Bononi A (2019). Mesothelioma: scientific clues for prevention, diagnosis, and therapy. CA Cancer J Clin.

[2] IARC (1977). Asbestos: IARC monographs on the evaluation of carcinogenic risks to humans.

[3] Chiappino G, Sebastien P, Todaro A (1991). Atmospheric asbestos pollution in the urban environment: Milan, Casale Monferrato, Brescia, Ancona, Bologna and Florence. Med Lav.

[4] Casali M, Carugno M, Cattaneo A, Consonni D, Mensi C, Genovese U (2015). Asbestos lung burden in necroscopic samples from the general population of Milan. Italy Ann Occup Hyg.

[5] Capella S, Bellis D, Fioretti E, Marinelli R, Belluso E (2020). Respirable inorganic fibers dispersed in air and settled in human lung samples: assessment of their nature, source, and concentration in a NW Italy large city. Environ Pollut.

[6] Oddone E, Bollon J, Nava CR, Bugani M, Consonni D, Marinaccio A (2020). Predictions of mortality from pleural mesothelioma in Italy after the ban of asbestos use. Int J Environ Res Public Health.

[7] Dodson RF, O’Sullivan M, Corn CJ, McLarty JW, Hammar SP (1997). Analysis of asbestos fiber burden in lung tissue from mesothelioma patients. Ultrastruct Pathol.

[8] Roggli VL, Sanders LL (2000). Asbestos content of lung tissue and carcinoma of the lung: a clinicopathologic correlation and mineral fiber analysis of 234 cases. Ann Occup Hyg.

[9] Churg A, Wright JL, Vedal S (1993). Fiber burden and patterns of asbestos-related disease in chrysotile miners and millers. Am Rev Respir Dis.

[10] Howel D, Gibbs A, Arblaster L, Swinburne L, Schweiger M, Renvoize E (1999). Mineral fibre analysis and routes of exposure to asbestos in the development of mesothelioma in an English region. Occup Environ Med.

[11] Churg A, Wiggs B (1986). Fiber size and number in workers exposed to processed chrysotile asbestos, chrysotile miners, and the general population. Am J Ind Med.

[12] McDonald JC, Armstrong BG, Edwards CW, Gibbs AR, Lloyd HM, Pooley FD (2001). Case-referent survey of young adults with mesothelioma: I lung fibre analyses. Ann Occup Hyg.

[13] Visonà SD, Crespi E, Belluso E, Capella S, De Matteis S, Filippi F (2022). Reconstructing historical exposure to asbestos: the validation of “educated guesses”. Occup Med.

[14] Selikoff IJ, Lee DHK (1978). Asbestos and disease.

[15] Visonà SD, Capella S, Bodini S, Borrelli P, Villani S, Crespi E, et al. Reply to Mirabelli et al. Is Mesothelioma Unrelated to the Lung Asbestos Burden? Comment on “Visonà et al. Inorganic Fiber Lung Burden in Subjects with Occupational and/or Anthropogenic Environmental Asbestos Exposure in Broni (Pavia, Northern Italy): An SEM-EDS Study on Autoptic Samples. Int. J. Environ. Res. Public Health 2021, 18, 2053.” Int J Environ Res Public Health. 2021 Jul 5;18(13). 10.3390/ijerph18137181 10.3390/ijerph18137181 PMC8297115 34281119

[16] Marinaccio A, Corfiati M, Binazzi A, Di Marzio D, Scarselli A, Ferrante P (2018). The epidemiology of malignant mesothelioma in women: gender differences and modalities of asbestos exposure. Occup Environ Med.

[17] Alpert N, van Gerwen M, Flores R, Taioli E (2020). Gender differences in outcomes of patients with mesothelioma. Am J Clin Oncol.

[18] Husain AN, Colby TV, Ordóñez NG, Allen TC, Attanoos RL, Beasley MB (2018). Guidelines for pathologic diagnosis of malignant mesothelioma 2017 update of the consensus statement from the international mesothelioma interest group. Arch Pathol Lab Med.

[19] Husain AN, Colby TV, Ordóñez NG, Krausz T, Borczuk A, Cagle PT (2009). Guidelines for pathologic diagnosis of malignant mesothelioma: a consensus statement from the international mesothelioma interest group. Arch Pathol Lab Med.

[20] Wolff H, Vehmas T, Oksa P, Rantanen J, Vainio H (2015). Asbestos, asbestosis, and cancer, the Helsinki criteria for diagnosis and attribution 2014: recommendations. Scand J Work Environ Health.

[21] Asbestos, asbestosis, and cancer: the Helsinki criteria for diagnosis and attribution. Scand J Work Environ Health. 1997; 23(4):311–6. 9322824

[22] Oddone E, Ferrante D, Cena T, Tùnesi S, Amendola P, Magnani C (2014). Asbestos cement factory in Broni (Pavia, Italy): a mortality study. Med Lav.

[23] Oddone E, Ferrante D, Tunesi S, Magnani C (2017). Mortality in asbestos cement workers in Pavia, Italy: a cohort study. Am J Ind Med.

[24] World Health Organization (2000). Regional Office for Europe. Air quality guidelines for Europe.

[25] Belluso E, Bellis D, Fornero E, Capella S, Ferraris G, Coverlizza S (2006). Assessment of inorganic fibre burden in biological samples by scanning electron microscopy—energy dispersive spectroscopy. Microchim Acta.

[26] Visonà SD, Capella S, Bodini S, Borrelli P, Villani S, Crespi E (2021). Evaluation of deposition and clearance of asbestos (detected by SEM-EDS) in lungs of deceased subjects environmentally and/or occupationally exposed in Broni (Pavia, Northern Italy). Front Public Health.

[27] Suzuki Y, Yuen SR, Ashley R (2005). Short, thin asbestos fibers contribute to the development of human malignant mesothelioma: pathological evidence. Int J Hyg Environ Health.

[28] Berman DW, Crump KS (2008). A meta-analysis of asbestos-related cancer risk that addresses fiber size and mineral type. Crit Rev Toxicol.

[29] Boulanger G, Andujar P, Pairon JC, Billon-Galland MA, Dion C, Dumortier P (2014). Quantification of short and long asbestos fibers to assess asbestos exposure: a review of fiber size toxicity. Environ Health.

[30] Gruppo Biofibre. Corpuscoli dell’asbesto nel tessuto polmonare umano e liquidi biologici: metodo analitico e atlante fotografico. Istituto Superiore di Sanità; 2017. Accessed 2 May 2023.

[31] Cooke WE (1929). Asbestos dust and the curious bodies found in pulmonary asbestosis. Br Med J.

[32] Oury TD, Sporn TA, Roggli VL (2014). Pathology of asbestos-associated diseases.

[33] Dodson RF, Hammar SP (2011). Asbestos: risk assessment, epidemiology and health effects. 6000 broken sound parkway NW, SUite 300.

[34] Visonà SD, Capella S, Bodini S, Borrelli P, Villani S, Crespi E (2021). Inorganic fiber lung burden in subjects with occupational and/or anthropogenic environmental asbestos exposure in Broni (Pavia, Northern Italy): an SEM-EDS study on autoptic samples. Int J Environ Res Public Health.

[35] Bernstein DM (2014). The health risk of chrysotile asbestos. Curr Opin Pulm Med.

[36] Churg A, DePaoli L (1988). Clearance of chrysotile asbestos from human lung. Exp Lung Res.

[37] Churg A (1994). Deposition and clearance of chrysotile asbestos. Ann Occup Hyg.

[38] Toyokuni S (2019). Iron addiction with ferroptosis-resistance in asbestos-induced mesothelial carcinogenesis: toward the era of mesothelioma prevention. Free Radic Biol Med.

[39] Churg A, Warnock ML (1977). Correlation of quantitative asbestos body counts and occupation in urban patients. Arch Pathol Lab Med.

[40] Case BW, Sebastien P, McDonald JC (1988). Lung fiber analysis in accident victims: a biological assessment of general environmental exposures. Arch Environ Health.

[41] Breedin PH, Buss DH (1976). Ferruginous (asbestos) bodies in the lungs of rural dwellers, urban dwellers, and patients with pulmonary neoplasms. South Med J.

[42] Rogers AJ, Leigh J, Berry G, Ferguson DA, Mulder HB, Ackad M (1991). Relationship between lung asbestos fiber type and concentration and relative risk of mesothelioma. A case-control study. Cancer.

[43] Sakai K, Hisanaga N, Huang J, Shibata E, Ono Y, Aoki T (1994). Asbestos and nonasbestos fiber content in lung tissue of Japanese patients with malignant mesothelioma. Cancer.

[44] Gilham C, Rake C, Burdett G, Nicholson AG, Davison L, Franchini A (2016). Pleural mesothelioma and lung cancer risks in relation to occupational history and asbestos lung burden. Occup Environ Med.

[45] Talc and Pyrophyllite Statistics and Information. https://www.usgs.gov/centers/nmic/talc-and-pyrophyllite-statistics-and-information. Accessed 6 Aug 2020.

[46] Gordon RE, Fitzgerald S, Millette J (2014). Asbestos in commercial cosmetic talcum powder as a cause of mesothelioma in women. Int J Occup Environ Health.

[47] Barbieri PG, Somigliana A, Chen Y, Consonni D, Vignola R, Finotto L (2020). Lung asbestos fibre burden and pleural mesothelioma in women with non-occupational exposure. Ann Work Expo Health.

[48] Consonni D, De Matteis S, Dallari B, Pesatori AC, Riboldi L, Mensi C (2020). Impact of an asbestos cement factory on mesothelioma incidence in a community in Italy. Environ Res.

[49] Marsh GM, Riordan AS, Keeton KA, Benson SM (2017). Non-occupational exposure to asbestos and risk of pleural mesothelioma: review and meta-analysis. Occup Environ Med.

[50] Xu R, Barg FK, Emmett EA, Wiebe DJ, Hwang WT (2018). Association between mesothelioma and non-occupational asbestos exposure: systematic review and meta-analysis. Environ Health.

[51] Roggli VL, Sharma A, Butnor KJ, Sporn T, Vollmer RT (2002). Malignant mesothelioma and occupational exposure to asbestos: a clinicopathological correlation of 1445 cases. Ultrastruct Pathol.

[52] Hodgson JT, Darnton A (2000). The quantitative risks of mesothelioma and lung cancer in relation to asbestos exposure. Ann Occup Hyg.

[53] Germine M, Puffer JH (2015). Analytical transmission electron microscopy of amphibole fibers from the lungs of quebec miners. Arch Environ Occup Health.

[54] Laaksonen S, Kettunen E, Sutinen E, Ilonen I, Vehmas T, Törmäkangas T (2022). Pulmonary asbestos fiber burden is related to patient survival in malignant pleural mesothelioma. J Thorac Oncol.

[55] Barbieri PG, Consonni D, Somigliana A (2022). Asbestos lung burden does not predict survival in malignant pleural mesothelioma: a necropsy-based study of 185 cases. J Thorac Oncol.

[56] Huang J, Chan SC, Pang WS, Chow SH, Lok V, Zhang L (2023). Global incidence, risk factors, and temporal trends of mesothelioma: a population-based study. J Thorac Oncol.

[57] Walker-Bone K, Benke G, MacFarlane E, Klebe S, Takahashi K, Brims F (2023). Incidence and mortality from malignant mesothelioma 1982–2020 and relationship with asbestos exposure: the Australian mesothelioma registry. Occup Environ Med.

