# Supplementary material for: A postmortem case control study of asbestos burden in lungs of malignant mesothelioma cases
Source: J Transl Med. 2023 Dec 2;21:875. doi: 10.1186/s12967-023-04761-9 (PMC10693031; doi:10.1186/s12967-023-04761-9)
Supplement: Supplementary file 1 — Additional file 1: Table S1. Reliability of measurements performed in two different laboratories. [file 12967_2023_4761_MOESM1_ESM.docx]

| **SUBJECT N** | **Mean Length (μm) Lab 1** | **Mean Length (μm) Lab 2** | **MeanWidth (μm) Lab 1.** | **Mean Width (μm) Lab 2** | **Asbestos (n. of observed fibers)**  **Lab 1** | **Asbestos (n. of observed fibers) Lab 2** | **AB (n. of observed fibers) Lab 1** | **AB (n. of observed fibers) Lab 2** | **Chrysotile**  **(n. of observed fibers) Lab 1** | **Chrysotile**  **(n. of observed fibers) Lab 2** | **Crocidolite**  **(n. of observed fibers)**  **Lab 1** | **Crocidolite**  **(n. of observed fibers)**  **Lab 2** | **Amosite (n. of observed fibers) Lab 1** | **Amosite (n. of observed fibers) Lab 2** |
| --- | --- | --- | --- | --- | --- | --- | --- | --- | --- | --- | --- | --- | --- | --- |
| **16178** | . | 27.3 | . | 0.9 | 0 | 2 | 0 | 0 | 0 | 0 | 0 | 1 | 0 | 1 |
| **16198** | . | . | . | . | 0 | 0 | 0 | 0 | 0 | 0 | 0 | 0 | 0 | 0 |
| **16381** | 15.16 | 18.1 | 0.8 | 0.7 | 44 | 38 | 4 | 2 | 0 | 0 | 13 | 10 | 8 | 11 |
| **17467** | 25.2 | 27.5 | 0.5 | 0.6 | 7 | 10 | 0 | 1 | 0 | 0 | 3 | 4 | 4 | 3 |
| **17473** | 21.96 | 25.3 | 0.6 | 0.4 | 7 | 3 | 3 | 6 | 0 | 0 | 5 | 5 | 3 | 1 |
| **17633** | 24.68 | 16.4 | 0.9 | 0.7 | 5 | 6 | 2 | 1 | 0 | 0 | 3 | 1 | 2 | 3 |
| **P value (ANOVA for repeated measures)** | 0.9893 | | 0.2522 | | 0.6656 | | 0.8220 | |  |  | 0.4896 | | 0.6606 | |

| **SUBJECT N** | **Anthophyllite (n. of observed fibers)**  **Lab 1** | **Anthophyllite (n. of observed fibers)**  **Lab 2** | **Tremolite/Actinolite (n. of observed fibers)**  **Lab 1** | **Tremolite/Actinolite (n. of observed fibers)**  **Lab 2** |
| --- | --- | --- | --- | --- |
| **16178** | 0 | 0 | 0 | 0 |
| **16198** | 0 | 0 | 0 | 0 |
| **16381** | 11 | 5 | 12 | 12 |
| **17467** | 0 | 0 | 0 | 3 |
| **17473** | 0 | 0 | 0 | 0 |
| **17633** | 0 | 0 | 0 | 2 |
| **P value (ANOVA for repeated measures)** | 0.3632 | | 0.1852 | |

Table S1 – reliability of measurements performed in two different laboratories.
